# Interactions of Amyloid-β with Membrane Proteins

**DOI:** 10.3390/ijms22116075

**Published:** 2021-06-04

**Authors:** Benita Wiatrak, Janusz Piasny, Amadeusz Kuźniarski, Kazimierz Gąsiorowski

**Affiliations:** 1 Department of Pharmacology, Wroclaw Medical University, Mikulicza-Radeckiego 2, 50-345 Wroclaw, Poland; 2 Department of Basic Medical Sciences, Wroclaw Medical University, Borowska 211, 50-556 Wroclaw, Poland; kazimierz.gesiorowski@umed.wroc.pl; 3 Department of Prosthetic Dentistry, Wroclaw Medical University, Krakowska 26, 50-425 Wroclaw, Poland; amadeusz.kuzniarski@umed.wroc.pl

**Keywords:** Alzheimer’s disease, amyloid-β, NMDA receptor, PrP^C^ protein, Fyn kinase

## Abstract

In developing and developed countries, an increasing elderly population is observed. This affects the growing percentage of people struggling with neurodegenerative diseases, including Alzheimer’s disease. Nevertheless, the pathomechanism of this disease is still unknown. This contributes to problems with early diagnosis of the disease as well as with treatment. One of the most popular hypotheses of Alzheimer’s disease is related to the pathological deposition of amyloid-β (Aβ) in the brain of ill people. In this paper, we discuss issues related to Aβ and its relationship in the development of Alzheimer’s disease. The structure of Aβ and its interaction with the cell membrane are discussed. Not only do the extracellular plaques affect nerve cells, but other forms of this peptide as well.

## 1. β-Amyloid in the Development of Alzheimer’s Disease

β-amyloid is a peptide composed of 39–43 pairs of amino acids [1]. The 28 amino acid residues of Aβ are N-terminal ending, and C-terminal segments terminate another 12–14 residues. The amine terminals are placed extracellularly and anchored in the transmembrane space. The carboxyl ends show hydrophobic and fibrillogenic properties [2].

In patients with Alzheimer’s disease (AD), the soluble form, which appears in the secondary beta-sheet conformation, is deposited in the form of 3–4-kDa-long vascular plaques and deposits [3,4]. As a result of these deposits, calcium channel activity in the synapses is disturbed, disrupting nerve signal transmission. The deposition of an excess of this protein can damage the mitochondria, thereby increasing free radicals and cell death. Simultaneously, the inflammatory process is exacerbated by the presence of microglia and astrocytes [5].

The peptide effect depends on the concentration: in the range of 10^−10^–10^−8^ M, it shows neurotrophic properties, and above 10^-7^ M it is neurotoxic [3]. In vitro studies have demonstrated that dimers are three times more toxic than monomers [6]. Soluble oligomers (usually 10–100 kDa) are considered more cytotoxic than amyloid fibril aggregates [3,7,8]. Oligomers easily diffuse into neurons by endocytosis. They inhibit many neuronal activities, influence the classical synaptic plasticity model, and participate in amyloid fibers [8,9]. The “amyloid hypothesis” has been formulated and suggests that β-amyloid deposition initiates neurodegeneration in AD [10]. However, according to some recent statements the development of neurodegeneration in this particular disease should be redefined since it may originate in combined pathophysiological mechanisms [11,12].

## 2. Structure and Cascade of β-Amyloid Transformations

Aβ arises from the processing of one or more isoforms of the APP precursor protein formed depending on the APP gene located on the 21st pair of chromosomes [3]. APP protein is a transmembrane glycoprotein that influences the development of the nervous system and neuron growth. It is involved in cell adhesion and synaptic connections [13]; its basic isoforms are APP695, APP751 and APP770 [3,14]. The APP protein participates in neuroprotective and neurotrophic processes (growth and differentiation of neurites), affects cell adhesion, and interacts with the intercellular substance components, including collagen I, laminin, fibronectin, heparin sulfate and glycosaminoglycans; it also participates in the modulation of synaptic plasticity. This protein plays an important role in maintaining a constant intracellular calcium Ca^2+^ [3,15].

**Scheme 1:**
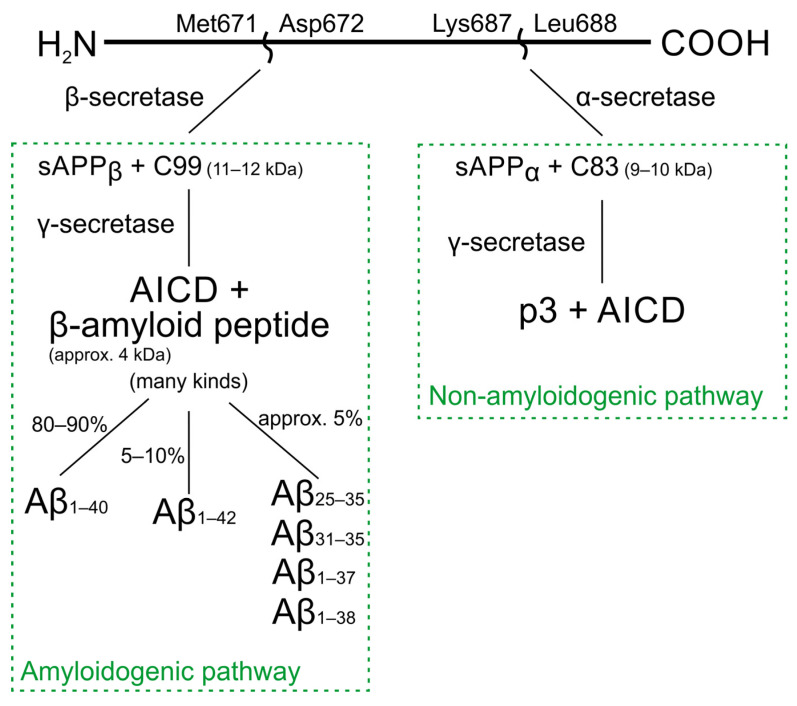
APP protein processing pathways.

There are two main APP processing pathways. Many extra- and intracellular factors can influence the way of processing. Furthermore, the mechanisms can be cell-, tissue- or species-specific. The APP protein is sequentially cleaved by the activity of proteolytic enzymes—secretases [16]. β- and γ-secretases participate in the amyloidogenic pathway, and α- and γ-secretases in the non-amyloidogenic pathway (Scheme 1) [16,17].

α-secretase, the activity of which increases after the activation of protein kinase C, reduces the proteolysis by β-secretase (amyloidogenic pathway) [16]. There are two main forms of the β-secretase enzyme—BACE1 and BACE2. The form responsible for the production of Aβ in the brain is primarily BACE1 [15]. γ-secretase is a protein complex composed of four components: presenilin (PSEN1 or PSEN2), nicastrin, and Aph1 and Pen2 proteins. The γ-secretase enzyme can only process substrates formed after the action of α- or β-secretase [16,18].

In the non-amyloidogenic pathway, cleavage of APP by α-secretases between Lys687 and Leu688 produces the soluble protein sAPPα and membrane-bound C83 peptide, which is then cleaved by γ-secretase into p3 and AICD fragments (APP intracellular domain) [19,20]. As a result of the stimulation of NMDA receptors with glutamate, the production of sAPPα (induction of the non-amyloidogenic pathway) increases.

In the amyloidogenic pathway, β-secretase cleaves APP between Met671 and Asp672, releasing soluble sAPPβ and the membrane-anchored peptide C99 [19]. The resulting amino acid is cleaved by γ-secretase to β-amyloid with a mass of approx. 4 kDa and AICD [21]. The action of γ-secretase can occur in different C terminal regions, resulting in several types of Aβ [16]. In a non-pathological process, mainly shorter Aβ_40_ is produced, which is usually about 80–90%. The longer form of Aβ_1–42_ is more hydrophobic and fibrillogenic [22,23].

Moreover, there is a less common path in which it is assumed that after APP cleavage, some Aβ remains in the cell membrane, forming Aβ oligomers, which affect the integrity of the cell membrane [24].

AD patients have increased production of Aβ_1–42_, which has a stronger tendency to accumulate as insoluble deposits compared to Aβ_1–40_. Furthermore, it has been observed that the carboxyl terminus (COO) of the APP protein may also be neurotoxic. APP processing is recognized as one of the key factors in the pathogenesis of Alzheimer’s disease [25].

**Scheme 2:**
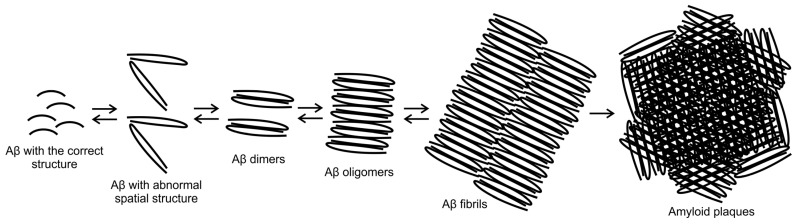
The process of the formation of senile amyloid plaques.

Senile plaques are mainly composed of Aβ_1–40_ and Aβ_1–42_. However, due to the proteolytic environment, shorter fragments are also formed (including Aβ_31–35_, Aβ_25–35_ and Aβ_27–42_), which are also present in smaller amounts in patients with AD [26]. Scheme 2 shows the process of the formation of senile plaques.

Aβ_25–35_, which shares many features with Aβ_1–40_ and Aβ_1–42_, is frequently used in in vitro experiments. In spectroscopic and physicochemical studies, the L and D stereoisomers of Aβ_25–35_ and Aβ_1–42_ proteins are similar. Immunohistochemical studies have shown Aβ_25–35_ in AD patients, while it was absent in the control group (healthy patients). Synthetic Aβ_25–35_ also displayed toxic activity in cell culture and ability to form aggregates with properties similar to deposits found in AD patients’ brains. It is often assumed that Aβ_25–35_ is a highly neurotoxic fragment of Aβ [26,27,28,29].

β-amyloid formed in the amyloidogenic pathway through β- and γ-secretases is also present in picomolar concentrations in people without cognitive impairment [30]. Soluble Aβ monomers are responsible for the growth of neurons and protection against oxidative stress and synaptic conduction modulation. They increase the expression of membrane potassium channels, resulting in significantly reducing apoptosis and excitability of neurons. Aβ binds to L-type calcium channels, increasing the level of Ca^2+^ inside the cell. Aβ monomers also improve Ca^2+^-dependent neuron conductivity [22,27,31]. Ion channels in the plasma membrane can influence the overall toxicity of Aβ by influencing its aggregation. The channels are believed to affect the aggregation of Aβ_1–42_ with no significant effect on Aβ_25–35_ [22,28].

## 3. Fragment of Aβ_25–35_ in the Pathomechanism of Alzheimer’s Disease

The formation of Aβ_25–35_ in the brains of elderly people and AD may be associated with the post-translational modification of senile amyloid proteins in the aging process of these long-lived proteins [28,32]. The aging of amyloid deposit proteins is accompanied by gradual racemization, mainly Ser and Asp, with D enantiomer appearance—this process takes about 20–30 years [32,33]. It has been shown that amyloid peptide racemization can also be caused by free radicals [34] and increased exposure to free radicals and chronic oxidative stress are important factors accelerating the development of neurodegeneration. The presence of D-enantiomers of the amino acid residues of serine and asparagine in the amount of approx. 5–10% of the total number of amino acids building Aβ in senile plaques was confirmed in AD patients [28]. The racemization of Aβ_1–40_ in the D residue of Ser^26^ is assumed to play an important role in AD pathogenesis [28,32,35]. Racemization of Ser^26^ leads to the formation of non-fibrillar, soluble [D-Ser^26^]Aβ_1–40_ from fibrillary, insoluble Aβ of amyloid deposits. Released from senile plaques, soluble [D-Ser^26^]Aβ_1–40_ has no neurotoxic properties. It is susceptible to protease attack with the formation of neurotoxic fragments, mainly [D-Ser^26^]Aβ_25–35/40_ [28,32]. Fragments of this peptide containing D-Ser^26^ show a high tendency to aggregate and are resistant to protease action, unlike other fragments generated in the degradation of Aβ [28,36]. Immunohistochemical studies of AD patients’ brains has confirmed the presence of [D-Ser^26^]Aβ_25-35_, while in the control group of healthy people of similar age, these fragments were not present [32].

It is likely that as a result of racemization, the [D-Ser^26^]Aβ_25–35_ fragment changes its conformation to the extent that hinders further proteolytic degradation and removal of this peptide. This could explain the higher content of this peptide in AD brains. The proteolytic degradation-resistant peptide [D-Ser^26^]Aβ_25–35_ may be considered an important source of the neurotoxic Aβ_25-35_ peptide in AD brains [28,32].

## 4. The Interaction of Aβ with the Cell Membrane Proteins

Extracellular Aβ, on its way to the cytosol of neurons, encounters the lipid bilayer barrier of cell membranes [37]. It is assumed that Aβ binds directly to membrane lipids, damages the lipid bilayer structure, penetrates cells, binds to receptors on cell membranes, and the receptor stimulation effect is transmitted in the cell through signal transduction pathways [37,38].

As a result of interactions with lipids of cell membranes, the conformation of amyloid peptides changes significantly—the share of the β structure increases and the formation of oligomers and their aggregates begins [37]. It is assumed that cell membranes lipid composition is of key importance for forming changes in the conformation of amyloid peptides, their biophysical features, including stiffness/elasticity and polarity, resulting from the specific composition of membrane lipids in the regions of interaction with Aβ [39,40]. The set of membrane lipids that Aβ encounters on its intracellular pathway forms the basis (matrix) for the formation of amyloid aggregates and fibers, and the binding of Aβ with cell membrane lipids induces its folding disorders (β-sheet structure) and oligomerization [22,38,41].

Electrostatic and hydrophobic interactions influence the attachment of Aβ to the cell membrane. Aβ is an amphiphilic peptide, the hydrophobic part is located at the C-terminus, and the hydrophilic part, at the N-terminus, contains 6 negatively charged amino acid residues (asparagine, glutamine) and 6 positively charged amino acids (lysine, arginine, histidine) [24,42]. In contact with cell membrane lipids, the conformation of amyloid peptides changes. The positively charged amino acids are exposed on the Aβ surface, which binds to the phospholipids of negatively charged cell membranes (sphingomyelin, containing phosphorus, and phosphoethanolamine in the hydrophilic head). It should be emphasized that the cell membranes show heterogeneity in the proportion of lipid components in different regions in the membrane plane; microdomains can be distinguished, differing in the proportion of various lipids and their physicochemical properties. A random event is whether Aβ particles encounter non-electrically charged lipid domains—electrostatically non-binding Aβ or negatively charged microdomains with which amyloid peptides can be electrostatically bound [43].

**Scheme 3:**
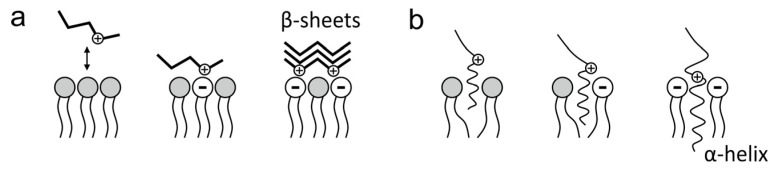
Electrostatic interactions of amyloid peptides with cell membrane lipids: (**a**) Aβ adsorption on the membrane surface; (**b**) Aβ implantation in the membrane.

The electrostatic interactions of Aβ and cell membrane lipids are shown in Scheme 3.

In interactions with membrane lipids showing a negative charge, the exposure of Aβ regions containing positively charged amino acid residues leads to changes in the peptide conformation, an increase in the proportion of the β-sheet structure and the formation of amyloid aggregates (Scheme 3a) [10,44]. In a segment of the membrane with a neutral potential, only a short part of the hydrophobic segment of Aβ becomes nested in the lipid bilayer, while when the negative charge increases (in a given region/microdomain of the membrane), the proportion of negatively charged phospholipids increases and the positively charged segments of Aβ are more attracted, leading to a stronger sinking segment of the hydrophobic amyloid peptide that adopts an α-helix conformation (Scheme 3b) [44].

Sphingomyelin-rich and cholesterol-rich regions/microdomains of lipid membranes play an important role in interactions with amyloid peptides. Sphingomyelin binds amyloid peptides on the cell membrane surface. It generates the initiation of the fibrilization process. At the same time, cholesterol present in the lipid bilayer’s deeper regions interacts with the amyloid peptide’s hydrophobic regions. It drags Aβ deeper into the lipid bilayer, enhancing conformational changes and amyloid peptide aggregation [45]. The optimal cholesterol content for Aβ binding and aggregation was determined to be about 35% in the sphingomyelin-rich cell membrane regions; then, the amyloid peptide binding capacity increases more than 2.5-fold compared to cholesterol-poor membranes [45]. Since the lipid bilayer enriched in sphingomyelin and cholesterol is present in the regions of microdomains, i.e., lipid rafts, it is assumed that the oligomerization and fibrilization of Aβ occur in rafts of cell membranes [45]. It has been shown that exogenously administered Aβ is concentrated in the lipid rafts of cell membranes and penetrates the cell’s interior through the rafts. Peptide integration in rafts may influence membrane damage and the progression of Alzheimer’s disease [46]. It has been shown that the aggregation of amyloid peptides on the membrane surface occurs only after saturation of the penetration path [47].

In vitro, Aβ may bind to sphingolipid derivatives (sphingomyelin or ganglioside GM1) and cholesterol [47]. However, considering the physiological proportions of the cell membrane’s lipid composition, sphingomyelin and cholesterol, not GM1 ganglioside, binds Aβ and induces changes in its conformation and aggregate formation [1]. Numerous previous works have highlighted the key role of GM1 in Aβ aggregation [48,49,50]. However, they were carried out in vitro at high, non-physiological concentrations of ganglioside times exceeding the average concentration of GM1 in gray matter and white brain tissue [51]. It is currently assumed that GM1 at physiological concentrations inhibits amyloid peptides’ binding to sphingomyelin and the oligomerization of Aβ and has neuroprotective properties—its effect on improving memory in the rat AD model has been shown [52]. Its beneficial effect is probably due to its neuroprotective and neuroregenerative properties [53].

**Scheme 4:**
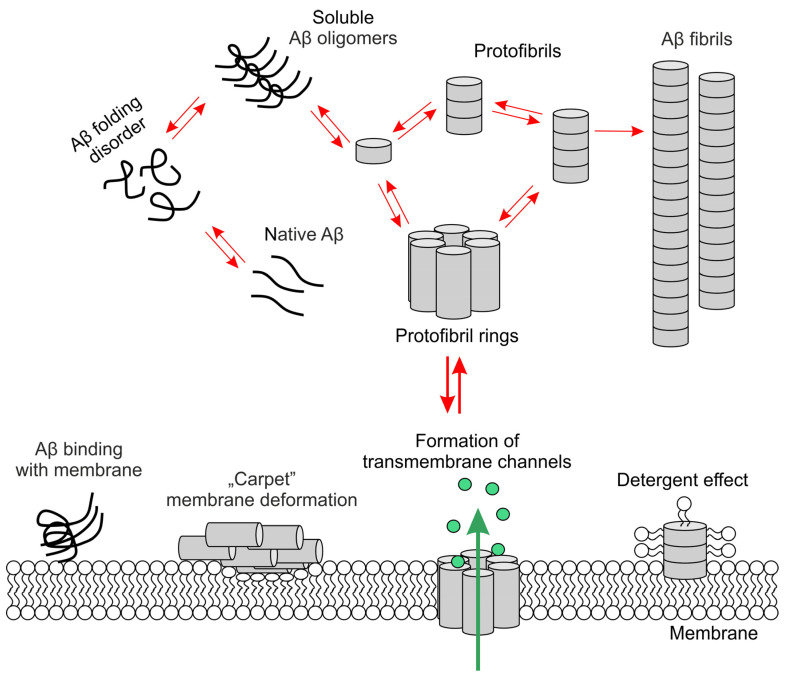
β-amyloid oligomers and aggregates in interactions with lipids in cell membrane.

According to Penke et al. [7], mature long amyloid fibrils may not be responsible for the toxic effects and damage to neurons’ key functions but the intermediate forms, oligomers and protofibrils, occurring during amyloid aggregation, may be responsible. In a model proposed by Bharadwaj et al. [41], transformations of native Aβ (monomers), i.e., changes in conformation, nucleation and elongation into oligomers, and then into protofibrils (including protofibril rings), are two-way processes (Scheme 4). Therefore, amyloid oligomers may be released during the protofibril fragmentation. Further fibrillization processes, i.e., the formation of amyloid aggregates from protofibrils, is an irreversible stage [54,55].

Oligomers and protofibrils interact with cell membranes in various ways, causing synaptic and neurotoxic effects [37]. After reaching the cutoff level, protofibrils may bind to the membrane, causing its thickness to decrease, e.g., by “carpet” deformation of the membrane or detergent effect (extraction of lipids from the membrane and incorporation into the amyloid fiber). In turn, protofibril rings can become embedded in the membrane, which leads to the formation of transmembrane channels that facilitate the migration of ions and water through the membrane; then the intracellular influx of Ca^2+^ increases, which leads to excitotoxicity and neuronal death due to the activation of calcium-dependent proteolytic enzymes [38,41].

In the case of the strongly neurotoxic fragment of the amyloid peptide, Aβ_25–35_, the neuronal membrane’s lipid composition is less important for aggregation, probably due to the high fibrilization rate of this amyloid fragment [27,56]. In studies on a model of a lipid membrane composed of phospholipids and cholesterol using nuclear magnetic resonance (NMR) spectroscopy, Aβ_25–35_ retained the same β-sheet structure regardless of the proportion of model lipids and formed transmembrane channels faster and in greater number compared to the full Aβ_1–42_ peptide [22,56]. An important ion channel in the cell membrane is the system of pumps and channels ensuring cellular homeostasis of Ca^2+^ ions. Ion transport to cells is mediated by voltage-gated Ca^2+^ channels (VGCC), including L-type, P-type and N-type channels. In Alzheimer’s disease, the important channel is the L type, which is stimulated by Aβ [57].

It has been shown that exogenous Aβ accumulates in large amounts in synaptic spaces, which suggests the involvement of membrane receptors, especially densely packed at synapses, in the binding of Aβ. Binding of Aβ with receptors present in synaptic membranes causes the activation effect of a given receptor (opening ion channels or activation of specific signal transduction pathways in ionotropic and metabotropic receptors, respectively), but also, in the case of some receptors, leads to endocytosis of the receptor–Aβ complex and increases intra-neuronal content of amyloid peptides [47]. Synaptic receptors for which Aβ receptor endocytosis has been demonstrated include: NMDA and AMPA glutamatergic receptors, α7nChR cholinergic receptor, LDLR and LRP1 lipoprotein receptors, and RAGE advanced glycation endproducts receptor [47]. The mechanism of Aβ binding to the ionotropic glutamatergic receptor NMDA and AMPA is relatively well known [39,47]. It has been shown that NMDA receptor endocytosis increases after the binding of extracellular Aβ, and the use of an antagonist of this receptor prevents the accumulation of amyloid in cells [47]. However, it has not been definitively confirmed whether Aβ binds directly to the NMDA receptor or whether another Aβ binding receptor mediates Aβ penetration. For example, Aβ binds to integrins of neuronal membranes, and an amyloid–integrin–NMDAR complex may be formed, which is endocytosed. Studies on the human neuroblastoma cell line have shown that integrin α5β1 participates in the penetration of soluble Aβ into cells, which intensifies its degradation and reduces neuronal apoptosis (there are fewer amyloid peptides in neurons and their surroundings) [58]. NMDA receptors and integrins have been suggested to cooperate to transport Aβ to the synapses of nerve cells [47]. The AMPA receptor, also found in lipid rafts, may be more effective than the NMDA receptor. After binding/activation by Aβ oligomers, the AMPA–Aβ receptor complex is rapidly endocytosed, and receptor inhibition stops amyloid entry into cells [47]. The Aβ also exhibits affinity for the insulin receptor [59].

In cases of Aβ receptor endocytosis, the complex is transported to endolysosomes where it undergoes proteolytic degradation. A necessary condition for the lysosomal proteolysis of endosomal cargo is the lysosomal matrix acidity (pH = 4.5–5.0). However, with age, the proton pump (V ATPase) function in the membranes of lysosomes and endosomes decreases, causing an increase in pH. The activity of lysosomal hydrolases decreases or even disappears [60]. In this case, the endosomal cargo is not digested in the lysosomes, and the Aβ oligomers may be released into the cytosol [60]. 

Binding of Aβ to the RAGE receptor (advanced glycation product receptor) also leads to the RAGE–Aβ complex’s endosomal internalization. However, the intracellular pathways of these endosomes lead to mitochondria, not lysosomes. Aβ penetrates from the endo-lysosomal compartment into the cytosol, from where a specific protein, the translocase, imports it into the mitochondria [47].

**Scheme 5:**
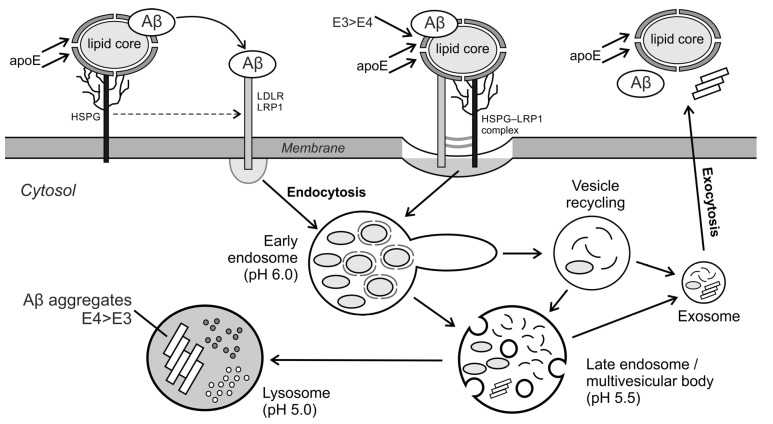
Aβ receptor endocytosis after binding to lipoprotein receptors: LDLR, LRP1 and/or heparan sulfate (HSPG) and the participation of apolipoprotein E in this process.

The receptors that bind Aβ oligomers with high affinity include the membrane lipoprotein receptors: LDLR, LRP1 and heparan sulfate (HSPG) proteoglycans anchored in cell membranes. β-amyloid (and especially oligomers) binds directly to these membrane receptors and is internalized by receptor endocytosis [61]. Many different coreceptors interact in endocytosis of the Aβ–LRP1 complex, including the membrane prion protein PrP^C^, HSPG9 [54], the cholinergic nicotinic receptor α7nAChR [61] and the cholinergic nicotinic receptor α7nAChR [47]. HSPG has been shown to bind Aβ with high affinity and then interacts with the LRP1 receptor to transmit the amyloid peptide to it. In this case, the LRP1–Aβ complex or the large HSPG–LRP1–Aβ complex is endocytosed [61]. Apolipoprotein E (ApoE) plays an important role in endocytosis involving the LRP1 receptor, which has high-affinity binding domains for Aβ (mainly oligomers), HSPG and LRP1 [61]. The fibrillogenesis pathways of Aβ oligomers depend on the ApoE isoform to which the amyloid peptide has bound. It was found that ApoE4 forms less stable complexes with Aβ (which may be due to the lower lipid content of the apolipoprotein E4 complex), shows a lower affinity for the LRP1 receptor and a lower rate of receptor endocytosis than in the case of the Aβ–ApoE3 complex [61]. As a result, receptor endocytosis and lysosomal degradation of Aβ oligomers in complex with ApoE4 are lower. At the same time, ApoE4 stabilizes extracellular amyloid oligomers, increases their aggregation and enhances senile plaque growth more strongly than ApoE3 [61]. The participation of ApoE in the binding of Aβ to LDLR receptors, LRP1 and HSPG proteoglycan, and the subsequent receptor endocytosis, is shown in Scheme 5.

After entering the cell by endocytosis, Aβ, as well as ApoE and LRP1, dissociated in the early endosome, are transported in the late endosomes to lysosomes, where they are degraded or are excreted from the cell in the exosomes via an alternative transport pathway (Scheme 5). For Aβ, the lysosomal transport pathway and proteolytic degradation prevail, although a small amount of Aβ is recycled and exocytosed outside the cell [61]. At a high concentration of Aβ in lysosomes, aggregation of Aβ can be initiated in the acidic lysosomal matrix (pH = 5.0), which may damage the lysosomes and release toxic aggregates, oligomers and protofibrils, into the cytosol of cells [61]. It should also be emphasized that with age, the efficiency of lysosomal proton pumps decreases and the lysosomal matrix’s pH is higher than optimal for the functions of lysosomal hydrolases [60]. Thus, it can be assumed that in the elderly, the share of the transport pathway for non-lysosomal degradable endosomes is much greater. For example, aggregation of amyloid oligomers was observed in early endosomes at pH ≈ 6–7 [62]. If the lysosomal pH is higher than optimal (pH > 5.0), the content of such vesicles is not degraded in lysosomes. The endosomes then form recycling vesicles and then exosomes, releasing the amyloid aggregates they contain to the outside of the cells, which leads to the growth of senile plaques. Some in silico models postulate defective autophagy of Aβ as an important pathological factor in the formation of amyloid deposits [63].

**Scheme 6:**
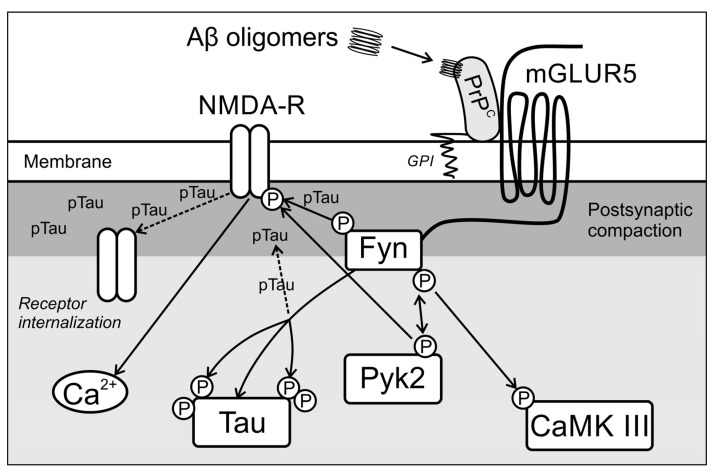
Intracellular signal transduction upon activation of cellular PrPC by Aβ oligomers.

The normal cellular prion protein PrP^C^ is a membrane receptor showing a particularly high binding capacity for Aβ oligomers [64], which occurs physiologically in the membranes of most types of cells, including synapses of neurons. After binding Aβ, the PrP^C^ protein does not cross the cell membrane; the activation effects by amyloid oligomers result from the activation of intracellular signal transduction pathways, as shown in Scheme 6.

The intracellular pathway of prion activation after binding to Aβ oligomers involves the activation of the non-receptor tyrosine kinase Fyn, mediated by the mGLUR5 transmembrane metabotropic glutamatergic receptor [64]. Fyn kinase regulates the phosphorylation and functions of the NMDA receptor and the tau protein [65]. Histological analysis of brains from AD patients confirmed the relationship between elevated levels of Fyn in neurons and hyperphosphorylated tau protein [64]. It has been shown that the physiological regulation of NMDA receptors by Fyn requires the presence of a functional tau protein, which is also a prerequisite for the Aβ-induced excitotoxicity of neurons [66]. NMDA receptor activation leads to increased Ca^2+^ influx into the cells and associated excitotoxicity. After the administration of exogenous Aβ, the increase in Fyn kinase activity is short-lived.

In contrast, the activation of Pyk2 (protein tyrosine kinase 2) and CAMK III (Ca^2+^/calmodulin dependent kinase III) kinases occur simultaneously, which participate in the mechanisms of synaptotoxicity and neurotoxicity [64]. Since PrP^C^ protein has a high affinity and the ability to bind Aβ oligomers, this pathway likely plays an important role, especially in the early stages of neurodegeneration when amyloid peptide levels are slightly elevated. Activation of Fyn kinase and Pyk2 and CAMK III kinases leads to increased phosphorylation of tau and the NMDA receptor, which destabilizes the receptor’s anchoring in the structures of postsynaptic densities [64]. The weakening of NMDA interactions with PSD-95, the main protein scaffolding postsynaptic densities, significantly impacts lowering LTP [64]. Scheme 6 illustrates the early, synaptic toxic effects of Aβ oligomers. On the other hand, higher concentrations of oligomers lead to increased binding to PrP^C^, increased NMDAR phosphorylation, the greater opening of the ion channel of this receptor, and increased excitotoxicity due to increased excitotoxicity to a significant influx of Ca^2+^ to neurons. Aβ–pTau–Fyn interactions are viewed as a toxic synaptic triad in the pathomechanism of AD [67]. Synaptic dysfunction can be induced by Aβ binding to receptors located in synapses, such as NMDAR, AChR and mGluR5 [47,67]. 

An important aspect is also the interaction with the membrane and receptors within the synapses. Apart from Aβ, other products of APP protein metabolism also participate in the interaction with receptors at synapses. Interaction of dissolved APP (sAPP) fragments with GABABR1a was demonstrated at synaptic terminals. GABABR1a receptors form heterodimers with GABABR2. The specific combination with GABABR1 results in inhibition of the release of synaptic vesicles, which consequently affects the suppression of synaptic transmission [68]. This interaction may lead to hyperphosphorylation of the tau protein. LilrB2 receptors also have high affinity (already in nanomolar concentrations) for Aβ oligomers in the brain, which can activate microglia. Its mouse counterpart (PirB) has been shown to affect synaptic plasticity. Another surface receptor that influences microglia activation is TREM2 [68]. ApoE binds to the TREM2 receptor and the Aβ binds them. Activated microglia may affect the persistent neuroinflammation in the brain, which deepens the progress of neurodegenerative processes.

Moreover, if there are redox-active metal ions in the intercellular space, Aβ can bind directly to metal ions, which can increase the level of free oxygen radicals and indirectly damage the cell membrane of neurons [69].

In conclusion, Aβ has been considered a cause of Alzheimer’s disease for many years. However, Aβ is a protein that is physiologically present also in healthy people. Due to lack of solid evidence it cannot be pointed out precisely which modifications of Aβ cause its toxicity. In this paper, we present an analysis of the interaction of various forms of Aβ with the proteins of cell membrane. As a result of this action, neurodegenerative processes deepen, both damaging the cell membrane as well as affecting the intracellular processes of neurons.

## 5. Research Methods Used to Analyze Interactions with Cell Membrane

When describing the interactions of amyloid with proteins of cell membranes, it is impossible not to mention the methods of studying their kinetics and spatial patterns. Interaction research methods can be divided into computational and experimental [70].

Computational models are an important tool in the study of neurodegenerative diseases since they are capable of simulating selected functions of nervous system cells and their reactions in different conditions and states [71]. Due to the development of computing power, the possibilities of conducting advanced analyses have increased, e.g., dynamic simulations of lipid environment influence, arrangement of lipid rafts and signalling pathways. Using computer modeling, it is possible to create 3D models of Aβ structures (monomers, oligomers, tetramers or octamers) and simulate their interactions with the cell membrane [24]. An important aspect in in silico studies is also the evaluation of membrane disruption mechanisms due to the interactions of Aβ and the cell membrane based on electrophysiology [24]. However, considering the complexity of neural processes in living organisms, these revelations should be treated with reserve as these models can often oversimplify actual mechanisms [71].

Commonly used experimental methods to study membrane interactions are nuclear magnetic resonance spectroscopy, mass spectrometry, fluorescence correlation spectroscopy and cross-correlation spectroscopy, super-resolution microscopy, Förster resonance energy transfer, membrane two-hybrid assays and photoactivatable or DNA-based lipid probes [70]. Nuclear magnetic resonance spectroscopy allows the analysis of the structure, dynamics, binding site and affinity of the protein–cell membrane complexes [72]. Mass spectrometry also provides the opportunity to study the dynamic interactions between a protein and the cell membrane [73]. However, unlike NMR, it only measures the mass and stoichiometry of the complex without being able to analyze the conformation shift. The disadvantage of this method is also the time-consuming analysis of the results—the larger the protein, the longer the analysis, which can take up to several days. Moreover, the need to use detergents in this method may also lead to misinterpretation of the obtained results, and interaction studies cannot be performed on living cells, contrary to NMR [70].

The method of fluorescence correlation spectroscopy, which allows the real-time study of cell membrane–ligand interactions, uses fluorescently labeled compounds that interact with the membrane [74]. The greatest disadvantage of this method is that it requires at least eight times the difference in molecular weight between the bound and unbound states [70]. A solution to this difficulty may be the use of fluorescence cross-correlation spectroscopy, which measures the correlation of two fluorescently labeled compounds [74]. However, both of these fluorescence spectroscopy methods are not suitable for monitoring slowly moving membrane proteins [70].

Super-resolution microscopy, in turn, allows the observation at the molecular level of cholesterol-assisted diffusion dependent on the distribution of sphingolipids and glycosylphosphatidylinositol and the formation of cholesterol-mediated complexes. This technique also enables the analysis of short-term transient interactions of compounds with the cell membrane [75].

The analysis of energy transfer between the donor and acceptor is possible using the Förster resonance energy transfer technique, and by combining with fluorescence microscopy this technique allows the analysis of transient interactions of the cell membrane [70].

In in vitro studies, colorimetric tests are commonly used methods that allow for multiple repetitions and are relatively cheap. In studies on the interaction of proteins/ligands with the membrane, they are used to assess the formation of bonds with the membrane, i.e., to which part of the membrane the protein binds, e.g., GM1 or cholesterol [49].

Membrane two-hybrid assays developed in yeast and mammals are a less popular but also used technique to analyze the interaction of proteins with cell membranes. Their advantage is the ease of use in high-throughput screening tests [76,77]. Photoactivatable or DNA-based lipid probes are also of great importance in screening. In the first case, photoactivatable probes are introduced into the cell membrane. Then, the cells prepared in this way are illuminated with light. Photoactive lipids bind to a ligand, including amyloid, which allows for interaction analysis. The advantage of this method is that it can be used in both living and artificial cell membranes [78]. In contrast, DNA-based probes can covalently modify membrane target lipids without affecting membrane integrity or integration into the membrane [78,79]. To analyze and interpret these interactions, among others, the techniques of spectroscopy and fluorescence microscopy are then used.

An interesting solution for analyzing the interaction of proteins with the cell membrane is also the use of techniques based on atomic force microscopy (AFM), e.g., high-speed AFM and nanoInfrared AFM. They allow spatial and temporal analysis of protein interactions, e.g., amyloid, with the cell membrane in real-time. Moreover, nanoInfrared AFM provides information on the chemical structure at the nanoscale without the labeling used in super-resolution microscopy [80].
